# Bacteria associated with vascular wilt of poplar

**DOI:** 10.1007/s00203-021-02464-7

**Published:** 2021-07-02

**Authors:** Hanna Kwaśna, Wojciech Szewczyk, Marlena Baranowska, Jolanta Behnke-Borowczyk

**Affiliations:** 1 Department of Forest Pathology, Poznań University of Life Sciences, Wojska Polskiego 71c, 60-625 Poznań, Poland; 2 Department of Silviculture, Poznań University of Life Sciences, Wojska Polskiego 71a, 60-625 Poznań, Poland

**Keywords:** Bacteria, Pathogens, Plantation, Poplar hybrids, Vascular wilt

## Abstract

In 2017, a 560-ha area of hybrid poplar plantation in northern Poland showed symptoms of tree decline. Leaves appeared smaller, turned yellow–brown, and were shed prematurely. Twigs and smaller branches died. Bark was sunken and discolored, often loosened and split. Trunks decayed from the base. Phloem and xylem showed brown necrosis. Ten per cent of trees died in 1–2 months. None of these symptoms was typical for known poplar diseases. Bacteria in soil and in the necrotic base of poplar trunk were analyzed with Illumina sequencing. Soil and wood were colonized by at least 615 and 249 taxa. The majority of bacteria were common to soil and wood. The most common taxa in soil were: Acidobacteria (14.76%), Actinobacteria (14.58%), Proteobacteria (36.87) with Betaproteobacteria (6.52%), (6.10%), *Comamonadaceae* (2.79%), and Verrucomicrobia (5.31%).The most common taxa in wood were: Bacteroidetes (22.72%) including *Chryseobacterium* (5.07%), Flavobacteriales (10.87%), Sphingobacteriales (9.40%) with *Pedobacter cryoconitis* (7.31%), Proteobacteria (73.79%) with Enterobacteriales (33.25%) including *Serratia* (15.30%) and *Sodalis* (6.52%), Pseudomonadales (9.83%) including *Pseudomonas* (9.02%), Rhizobiales (6.83%), Sphingomonadales (5.65%), and Xanthomonadales (11.19%). Possible pathogens were *Pseudomonas, Rhizobium* and *Xanthomonas.* The potential initial, endophytic character of bacteria is discussed. Soil and possibly planting material might be the reservoir of pathogen inoculum.

## Introduction

Poplars are distributed predominantly throughout the northern hemisphere. Because of their rapid growth, wild *Populus* spp. and their hybrids are currently planted over huge areas worldwide as ornamental plants for landscape greening, production of wood, and multiple industrial uses (Jansson and Douglas 2007).

Poplar’s susceptibility to phytopathogens is the main obstacle to its exploitation (reviewed in Newcombe 1996). The most serious fungal pathogens include vascular and parenchymal colonizers (Kwaśna et al. 2021).

There are also a few bacteria pathogenic on poplar. *Pseudomonas syringae* Van Hall and *Xanthomonas populi* (Ridé) Ridé & Ridé cause wilting, necrosis, rot, injury, tumours and cankers (Ridé 1993; Nesme et al. 1994; Yuki et al. 2013). Symptoms caused by *P. syringae* in combination with frost occur usually on the south side of trees, just above soil level, and the development of *P. syringae* in poplar bark is promoted by fluctuating temperatures (Ramstedt et al. 1994). *Pseudomonas aeruginosa* (Schröter) Migula appeared on poplar recently (Attila et al. 2008). It causes soft rot, which results in wilting, with death of trees occurring quickly, even in less than 48 h. *Rhizobium radiobacter* Beijerinck and van Delden [syn. *Agrobacterium radiobacter* (Beijerinck and van Delden) Conn, *Agrobacterium tumefaciens* (Smith and Townsend) Conn] causes crown gall disease by transferring and integrating bacterial DNA (T-DNA) into the plant genome. In the 1990s, the bacterial genus *Brenneria* was also reported to cause canker of trees including poplar (Biosca et al. 2006). Currently, large portions of the plantation areas of the hybrid poplar *Populus* × *euramericana* in China and Hungary are affected by potentially lethal *Lonsdalea populi* (formerly *Lonsdalea quercina* subsp. *populi*) (Toth et al. 2013; Li et al. 2014; Li and He 2019). Bacteria overwinter on infected plant tissues, in necrosis, gummosis and sap oozing from wounds or healthy looking plant tissues.

Bacteria spread by windblown water droplets, contaminated tools, insects and animals. Infection is through roots, leaf scars and fresh wounds on branches and stems. The extent of symptoms depends on tree susceptibility. Planting highly resistant clones from selections and breeding programs is the only way to control bacterial disease.

In 2017, 560 ha of plantation of hybrid poplar (*P. deltoides* × *P. nigra*) in northern Poland showed symptoms of tree decline. Leaves of diseased trees appeared smaller, turned yellow–brown, and were shed prematurely. Twigs and smaller branches died without definite cankers. Bark of the entire trunk was sunken and discolored, often loosened and split. It often fell off, exposing wet wood. Trunks decayed from the base. The phloem showed brown necrosis. Ten per cent of trees died in 1–2 months (in June) after the first appearance of symptoms. None of the observed symptoms was typical for known poplar diseases. The possible contribution of vascular and parenchymal fungal pathogens has been suggested (Kwaśna et al. 2021).

The objectives of this research were to study: (1) the abundance and diversity of bacteria in soil and wood that may possibly contribute to development of vascular wilt in poplar, and (2) interactions among these bacteria and environmental conditions.

## Materials and methods

### Site and sampling

The study was carried out in Łoża, Czarne District, Człuchów County, Pomeranian Voivodeship, northern Poland (53°41′29″ N 17°04′19″ E), in 560 ha of plantations of 5–6-year-old hybrid poplar (*P. deltoides* × *P. nigra*, cultivar AF2, from Italy) showing symptoms of crown decline and trunk-base decay (520 ha) and tree death (40 ha).

Trees were grown at a density of 425 trees/ha (4 × 4 m spacing), and had mean diameter of 9–10 cm at breast height. The post-agricultural soil was sandy loam, consisting of sand (60%), silt (20%) and clay (20%), with low humus level, and with pH 6.5. The former crop was rye (*Secale cereale* L.). The average temperature is 7.9 °C and rainfall 680 mm.

The understorey vegetation included *Achillea millefolium* L., *Agrostis stolonifera* L., *Artemisia absinthium* L., *Artemisia vulgaris* L., *Cichorium intybus* L., *Elymus repens* (L.) Gould*, Lamium purpureum* L., *Lolium perenne* L., *Papaver rhoeas* L., *Poa annua* L*., Poa pratensis* L., *Poa trivialis* L., *Polygonum aviculare* L., *Polypodium vulgare* L., *Polytrichum commune* Hedw., *Stellaria media* Hist. Pl. Dauphiné, *Taraxacum officinale* F.H. Wigg., and *Trifolium arvense *L.

Five wood cores, c.10 cm long and 3 cm diameter, each including bark, phloem and xylem, were sampled from the bases of necrotic trunks of five symptomatic trees, 0 cm and 50 cm above the soil surface, with a Pressler borer. The core samples were surface-sterilized before being ground to sawdust with a cordless SPARKY BUR2 15E drill. In addition, five subsamples of soil were taken as cylindrical cores, 10 cm long and 5 cm diameter, from the surroundings of roots of five symptomatic trees. They were placed in sterile glass containers and refrigerated for 48 h. There were no healthy, asymptomatic, trees in plantation which could be analyzed as the control.

### DNA extraction, amplification and Illumina sequencing

Five samples of sawdust were prepared from five wood cores in a SPEX™ SamplePrep™ Freezer/Mill™ cryogenic mill. Genomic DNA from wood was extracted in 100 mg of sawdust from each of the five wood cores with GeneJET™ Plant Genomic DNA Purification Mini Kit (Thermo Scientific, USA). Genomic DNA from soil was extracted in 300 mg of soil from each of the five soil cores with DNeasy PowerSoil Pro Kit (Qiagen, Hilden, Germany). The 16S rDNA from each subsample was amplified with bacteria-specific primers: 16S-F 5′ CAGCCTACGGGNGGCWGCAG and 16S-R 5′ ACAGGACTACHVGGGTATCTAATCC. Each amplification reaction was carried out in a final volume of 25.0 μL containing 2 μL DNA, 0.2 μL of each primer, 10.1 μL deionized water and 12.5 μL 2X PCR MIX (A & A Biotechnology, Gdynia, Poland). DNA amplification was performed under the following conditions: denaturation at 94 °C for 5 min, followed by 35 cycles of denaturation at 94 °C for 30 s, annealing at 56 °C for 30 s, elongation at 72 °C for 30 s, and a final elongation at 72 °C for 7 min. Visualization of 5-μl amplicons was performed in 1.0% agarose gel dyed with Midori Green Advance DNA (Genetics). Pooled PCR products were purified using a MinElute PCR Purification Kit (Qiagen, Hilden, Germany). The concentration of PCR products was quantified using a Qubit 2.0 Fluorometer (Life Technologies, Carlsbad, CA, USA), and an equimolar mix of PCR products from each sample was prepared. The PCR product from each subsample was purified and sequenced with the use of Illumina SBS technology (Genomed S.A. Warsaw, Poland).

### Bioinformatics analysis

A table of Operational Taxonomic Units (OTUs) was prepared by PIPITS, version 1.2.0 (Gweon et al. 2015). Read-pairs were joined with PEAR, version 0.9.6 (Zhang et al. 2014), filtered with a quality threshold of *q* = 30 by FASTX-toolkit, version 0.0.13 (http://hannonlab.cshl.edu/fastx_toolkit/index.html), converted to Fasta format and merged into a single file. Prepared sequences were de-replicated and subregions of ITS were selected with the use of ITSx, version 1.0.11 (Bengtsson-Palme et al. 2013). Unique sequences and those shorter than 100 bp were removed. Remaining sequences were clustered with 97% sequence identity. The resulting representative sequences for each cluster were subjected to chimera detection and removal using the UNITE UCHIME reference data set, version 6.0 (https://unite.ut.ee/index.php). The input sequences were then mapped onto the representative sequences and taxonomy assigned using RDP Classifier, version 2.10.2 (Wang et al. 2007), against UNITE fungal ITS reference database, version 11.2 (Cole et al. 2014). This process resulted in the creation of a table of OTUs. Sequences were identified by comparison with reference sequences from the National Center for Biotechnology Information (NCBI) database.

 Abundance of bacteria was defined as the average number of OTUs from five subsamples. Frequency of an individual taxon was defined as percentage (%) of OTUs in the total number of OTUs from five subsamples. Diversity of bacterial community (%) evaluated with the number of taxa in soil or wood is shown by a heatmap.

### Statistical analyses

Differences in abundance of bacteria in soil and wood were analyzed with Chi-squared tests (*χ*^2^). Diversity between communities of microfungi was compared with Margalef’s diversity index (*D*_Mg_), Shannon’s diversity index (*H*′), Simpson’s diversity index (*D*), Shannon’s evenness index (*E*) and Berger–Parker’s index (*d*) (Magurran 1988).

## Results

A total of 34,866 and 21,207 OTUs were obtained, respectively, from the soil and wood of *Populus* hybrid using the Illumina sequencing technique (Table 1). The majority of bacteria were classified to the higher taxa. Classification resulted from the length of the sequence. The frequency of classified and non-classified bacteria was 96.57% and 3.43% in soil and 99.84% and 0.16% in wood. The frequency of bacteria absent from NCBI database was 0.70% and 0.63% in soil and wood, respectively. Soil and wood were colonized by at least 615 and 249 taxa. There were 242 non-identified taxa in soil and 16 in wood. The majority of bacteria were common to soil and wood.

**Table 1:**
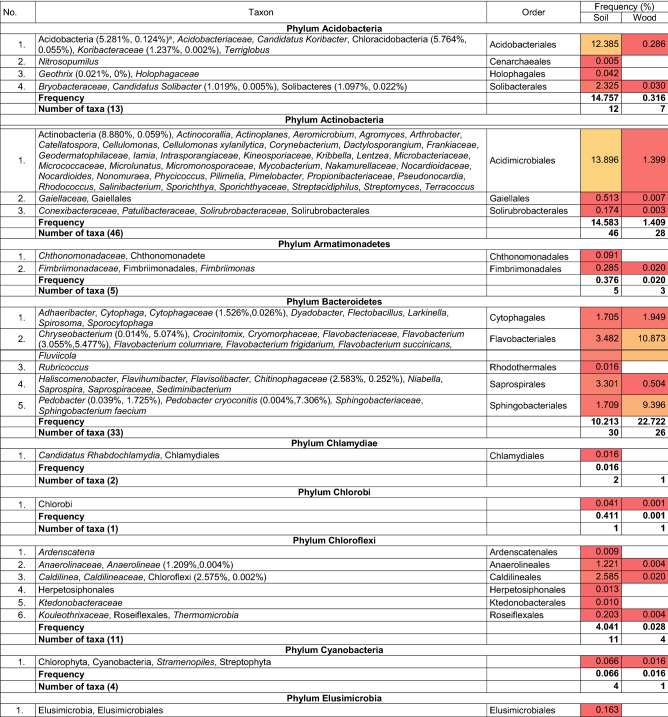

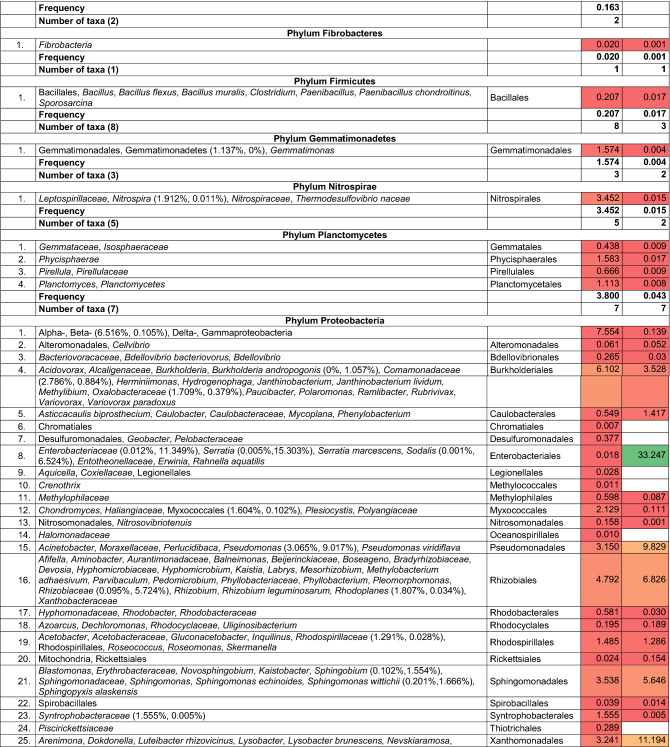

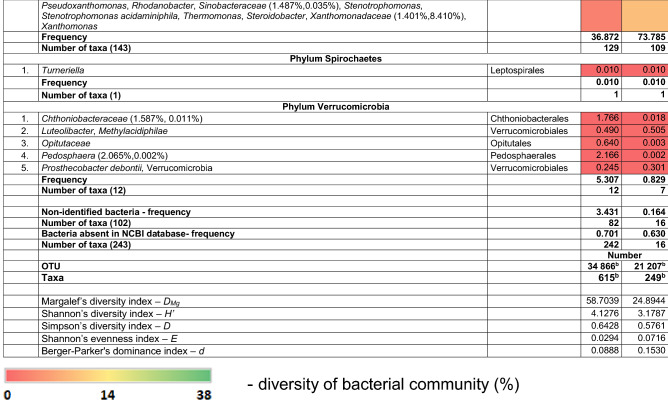
Bacteria present in soil and wood of wilted poplar

Explanations

^a^Indicates frequency in soil and wood, respectively

^b^Indicates a statistically significant difference according to χ^2^ test, *P *< 0.001

The most common taxa in soil were: Acidobacteria (with Acidobacteriales and Solibacterales), Actinobacteria (with Acidimicrobiales), Bacteroidetes (with Cytophagales, Flavobacteriales, Saprospirales and Sphingobacteriales), Chloroflexi (with Caldilineales)*,* Nitrospirae, Planctomycetes, Proteobacteria (with Betaproteobacteria, Burkholderiales, Pseudomonadales, Rhizobiales, Sphingomonadales and Syntrophobacterales) and Verrucomicrobia (with Chthoniobacterales and Pedosphaerales).

The most common taxa in wood were: Bacteroidetes (with Flavobacteriales and Sphingobacteriales), Proteobacteria (with Burkholderiales, Enterobacteriales, Pseudomonadales, Rhizobiales, Sphingomonadales and Xanthomonadales).

Most taxa were present in wood of the diseased poplars and in soil. Potential pathogens were species in genera *Pseudomonas* and *Xanthomonas.* Dubious and unexpected species include *Pedobacter cryoconitis* and *Sodalis.*

Margalef’s index (*D*_Mg_) and Shannon’s diversity index (*H*′) indicate greater diversity in soil than in wood, although Simpson’s diversity index (*D*) suggests more diversity in wood (Table 1). Conversely, Shannon’s evenness index (*E*) shows more evenness in communities in wood, although Berger–Parker’s dominance index (*d*) shows more dominance of individual taxa in wood.

## Discussion

Research on bacteria has usually been focused on the study of single species. However, bacteria are social organisms and they occur in communities. Recent advances in genomics and molecular techniques have led to discovery and characterization of a vast bacterial diversity and to understanding of mutual interactions among them.

Illumina sequencing of 16S rRNA genes was applied for studies of the bacteria associated with vascular wilt of poplar. Sequencing of the 16S rRNA gene is highly useful for bacterial classification because of its presence in almost all bacteria, its ability to exist as a multigene family or operon, its resistance to change over time, and its size (1500 bp long).

However, the 16S rRNA genes are highly conserved and do not provide sufficient resolution at lower taxon levels (i.e., species or strain). Thus, most bacteria in our studies were classified to the higher taxonomic ranks, making them ecologically and functionally indistinguishable. More precise taxonomic resolution was not possible because of: (1) insufficient number of sequences of the lower taxa in nucleotide databases, (2) occurrence of species sharing similar and/or identical 16S rRNA sequences, (3) nomenclature problems arising from multiple genomovars assigned to single species or complexes, and (4) the possible occurrence of new taxa. These reasons undoubtedly affected the scientific completeness of the research presented. Therefore, in many cases, the data are discussed and interpreted with caution and conclusions formulated with care. Only assumptions can be made.

In wood of diseased poplar the most common taxa of bacteria were: Bacteroidetes, particularly *Chryseobacterium* and *Flavobacterium*; Proteobacteria including Enterobacteriales, particularly *Serratia* and *Sodalis*; Pseudomonadales; Rhizobiales; Sphingobacteriales including *Pedobacter cryoconitis*; Xanthomonadales.Each of these taxa includes species or strains with various trophisms and functions: endophytes, pathogens or saprotrophs. It is not easy to draw a clear distinction between pathogens and non-pathogens. They occupy the same ecological niches and possess similar mechanisms for plant colonization. Endophytic, pathogenic and saprotrophic strains are often found within the same species, and the incidence and severity of potential diseases are affected by additional factors, including host vigour, environmental conditions and host–pathogen specific interactions (Schulz et al. 1999).

Members of *Chryseobacterium* have so far been found in various environments, i.e., freshwater, soil and sludge, rhizosphere and phyllosphere, midgut of insects, faeces of millipede, raw fish, chicken and dairy products, or clinical samples. On poplar, *Chryseobacterium* has been detected on two hybrid clones, where it was classified as an endophyte (Ulrich et al. 2008).

Flavobacteria are generally known as common, free living organisms in soil and water. Most species are harmless, psychrotolerant, present in temperate to polar regions, and particularly in low salinity ecosystems (Kolton et al. 2016). Several species are infectious to freshwater fish and humans (Berg et al. 2005). Little is known about the occurrence of *Flavobacterium* in plants. The first evidence of *Flavobacterium* in plants was from a study of the barley rhizosphere (Johansen et al. 2002). Recently, Kolton et al. (2016) detected epiphytic and endophytic Flavobacteria on/in roots and leaves of cucumber, lettuce, maize, peanuts, peppers, tomatoes, thale cress [*Arabidopsis thaliana* (L.) Heynh.] and wheat*.* They may be associated with plant health. They move rapidly over solid surfaces due to unique gliding-motility (linked to a novel type IX secretion system), enabling fast propagation and colonization (McBride and Zhu 2013; McBride and Nakane 2015). *Flavobacterium johnsoniae* causes a ‘soft-rot’ of various fresh plants (Liao and Wells 1986). *Flavobacterium* abundance can vary considerably and is a function of plant and environment interactions. So far there was no information on occurrence of *Flavobacterium* in forest habitats. Our record is the first. Its occurrence could possibly have resulted from the slightly less acid soil (pH 6.5) of the diseased poplar plantation.

Enterobacteriales are facultative anaerobes, common in water or soil, or are parasites of animals and plants. They can live on/in wood of poplar, and even much longer than on wood of other tree species (Schönwälder et al. 2002; Milling et al. 2005). The most abundant representatives of Enterobacteriales were *Serratia* and *Sodalis* (15.3% and 6.5% in wood). *Serratia* is often a harmless, environmental, plant-associated endophyte or free-living bacterium, found in decaying plants or animals and humans. Many *Serratia* species have plant growth-promoting (PGP) ability and have been developed as biocontrol agents for soil-borne fungal pathogens (Hallmann et al. 1997).

The very fast development of wilt in the poplar trees studied suggests that pathogenic *Pseudomonas*, i.e., *P. syringae* and *P*. *aeruginosa*, may be involved in development of disease. *Pseudomonas* occurred relatively abundantly in soil from where they could infect roots. The short span of the disease in the surveyed poplars may also have resulted from a quick response of the host plant. It is known that perception of *Pseudomonas* by plants occurs quickly. Changes in plant signal transduction and in plant gene expression occur within 2 and 15 min, respectively, after infection and exposure to bacterial elicitors (Gómez-Gómez et al. 1999; de Torres et al. 2003). The first cellular symptoms are observed within 5 h (Bestwick et al. 1997). Bacteria live on nutrients present in the apoplast of host cells, the acidic components of cell walls and nutrients in dying cells (Preston 2004). Apart from changes in the plant’s physiology, *Pseudomonas* can cause mechanical damage of the host tissues through the ice nucleation (Wisniewski et al. 1997). They may contribute to the final disease effect. The second potentially pathogenic species, *P. aeruginosa*, is an environmental organism that can survive in different conditions and is particularly well-adapted to wet and damp habitats, i.e., soil, aqueous solutions (Jefferies et al. 2012), and thus probably also in sap oozing from wounds on the diseased poplars. It forms biofilms which increase its persistence and stability and generate extensive genetic diversity which enables the bacteria to persist and spread under different environmental stresses (Webb et al. 2003). The genus *Pseudomonas* may also include neutral or beneficial strains, i.e., with biocontrol- or plant growth-promoting activity, or capable of inducing systemic plant defense. One of those is *Pseudomonas fluorescens* Migula, usually very common in soil and rhizosphere, and on plant surfaces (Silby et al. 2009). Degradative enzymes determine the virulence of pathogenic strains (Preston 2000). However, the same enzymes are produced by plant growth-promoting *Pseudomonas.* Thus, distinguishing between pathogenic and beneficial interactions needs recognition of: (1) the specificity and combination of extracellular compounds produced by the bacterium, (2) effects of habitat conditions (temperature, moisture) on host and bacterium, (3) host genotype, (4) host physiology, (5) the ability of the bacterium to respond to host recognition (Preston 2004). Success for the plant depends on its ability to distinguish beneficial symbionts and harmless saprotrophs from pathogenic parasites, and in using induced defense responses to eliminate dangerous pathogens at minimum cost.

Rhizobiales are well-known, beneficial, microsymbiotic, legume-nodulating, nitrogen-fixing, methanotrophic bacteria providing nutrients, vitamins, phytohormones (auxins and cytokinins) and precursors of essential plant metabolites (Vorholt 2012).

Nitrogen fixation by Rhizobiales has recently been found also in non-leguminous plants (Fischer et al. 2012), including wild poplar harbouring diazotrophic bacteria. However, poplar can be a natural host of pathogenic *R. radiobacter*, the occurrence of which in the microbiome of the studied poplars cannot be excluded, although the typical symptoms of *R. radiobacter*, i.e., tumour-like growths (galls) on stems and roots, often above ground, were not observed.

Sphingobacteriales are common in soil and marine habitats. One member of this order, *Flavitalea populi*, has been isolated from soil of a Euphrates poplar (*Populus euphratica* Oliv.) forest (Wang et al. 2011).

Xanthomonadales are typically rod-shaped, obligate aerobes with optimal growth at 25–30 °C. There are at least 27 plant-associated *Xanthomonas* species, which colonize at least 400 plant species (An et al. 2019). *Xanthomonas populi* can cause wilting, necrosis, rot and injury of poplar. It initially feeds on living host tissue and kills the tree in later stages of infection. Xanthan, a biopolymer, contributes to formation of biofilm which masks the bacteria, preventing recognition and early response from plant defense mechanisms (Büttner and Bonas 2010).

Betaproteobacteria, common in the soil samples, occupy diverse habitats and have various metabolic strategies. They may be autotrophic, heterotrophic and diazotrophic. Some, including *Bordetella, Burkholderia* and *Ralstonia*, are pathogenic on plants (Dworkin et al. 2006). However, the volatile organic compounds of *Burkholderia pyrrocinia* strain JK-SH007 inhibit three poplar canker pathogens: *Cytospora chrysosperma* (Pers.) Fr., *Phomopsis macrospora* Tak. Kobay. and Chiba, and *Fusicoccum aesculi* Corda) (Liu et al. 2020).

Dubious and unexpected species include *Pedobacter cryoconitis* (7.306% in wood) and *Sodalis* (6.524% in wood)*. Pedobacter cryoconitis* is a rod-shaped bacterium, facultatively aerobic and psychrophilic. Other *Pedobacter* species have been isolated from soil and compost, water and freshwater-lake sediment, and plant rhizosphere (Kwon et al. 2007, 2011). So far, *Pedobacter* was not found in the forest habitat. However, the bacterium is a cellulose decomposer, and this explains its abundant occurrence in wood. Endosymbiontic *Sodalis* has so far been associated with spittlebugs and feather-chewing bird lice, and a human wound.

Many bacteria detected were possibly the initial endophytes in a variety of tissues in trees that were, so far, healthy. They probably do not participate in the initial development of disease but become pathogenic later, after development of disease started by fungi (Kwaśna et al. 2021). They need favorable conditions, including lower temperature and higher humidity of soil from where they spread to plants. Their adaptation strategies for growth, production and activation of enzymes could compensate for the occasionally negative effects of the habitat.

Plants can be colonized simultaneously by a large variety of bacteria (Bacon and Hinton 2006). Most studies on bacterial endophytes were done on agricultural and horticultural plants. However, endophytic bacteria have also been detected in trees: elm, pine, oak, citrus and coffee (Bacon and Mead 1971; Mocali et al. 2003; Vega et al. 2005). Their survival was strongly dependent on moisture.

Endophytic bacteria in poplar have rarely been studied. Single studies have described only specific strains (Germaine et al. 2004; Van Aken et al. 2004; Doty et al. 2005). More recently, Moore et al. (2006) and Ulrich et al. (2008) studied the diversity of endophytic bacteria in *Populus* growing in contaminated and non-contaminated fields. They reported a high diversity and domination of Gammaproteobacteria (28–61%), especially *Pseudomonas* spp. (19–46%). In the present study *Pseudomonas* frequency was 3–9%, and other Gammaproteobacteria were sporadic in soil (0.01%) and absent in wood. The differences in abundance and diversity may result from the genetic background of the host tree (Ulrich et al. 2008). Each tree species or clone has an association with specific bacterial endophytes (Cambours et al. 2005; Moore et al. 2006) and the dependence of endophytic bacterial communities on host genotype seems to be stronger in longer-lived trees. In the endophytic phase, bacteria may have beneficial effects on their hosts and may play an important role in plant physiology, including resistance. Apart from *Pseudomonas*, other bacteria detected in soil or wood of the poplars studied, i.e., Actinobacteria*, Bacillus, Burkholderia* and *Chryseobacterium*, promote plant growth by elimination of pathogenic microorganisms, synthesis of growth-stimulating plant hormones, low-molecular compounds or enzymes, and creation of plant disease resistance, particularly during periods of drought or nutrient deprivation (Shin et al. 2007; Montero-Calasanz et al. 2013; Timm et al. 2016). Their presence in soil is explained by their ability to live on organic debris as saprotrophs. They usually colonize the host plant from the rhizosphere soil. Their adaptation to an endophytic or pathogenic lifestyle in plants results from their ability to pass (actively or passively) through the endodermis and pericycle and enter the xylem vessels (Compant et al. 2010).

## Conclusions

New diseases, including wilt, necrosis of stems and dieback may appear in forests and plantations of trees. *Populus* hybrids may be subjected to various, so far unidentified pathogenic agents, including bacteria. Bacteria can contribute to the development of disease, but can also have an important role in limiting or preventing the development of initial pathogens. This situation can lead to near-total disappearance of some diseases and sudden emergence of new pathogens. Poplar wilt symptoms may be a consequence of various factors, the most important being climate and its effects on development of pathogens and the host–pathogen relationship. New diseases can spread from soil or from introduced plant material, the latter potentially introducing them into new areas.

## Data Availability

Data associated with a paper is available at https://figshare.com/s/dd06afb0fb7d348f5388?fbclid=IwAR15_ FXPrsPve6TSo4cBUFtNLR655ZE6FFjpK1X80TVr706IDDTb6dVcco.
